# Supplementary material for: Polarized NHE1 and SWELL1 regulate migration direction, efficiency and metastasis
Source: Nat Commun. 2022 Oct 17;13:6128. doi: 10.1038/s41467-022-33683-1 (PMC9576788; doi:10.1038/s41467-022-33683-1)
Supplement: Supplementary file 1 — Supplementary Information [file 41467_2022_33683_MOESM1_ESM.pdf]

Supplementary Information file for:

Polarized NHE1 and SWELL1 Regulate Migration Direction, Efficiency and Metastasis

Yuqi Zhang[1,2], Yizeng Li[3], Keyata N. Thompson[4], Konstantin Stoletov[5], Qinling Yuan[1,2], Kaustav Bera[1,2], Se Jong Lee[1,2], Runchen Zhao[1,2], Alexander Kiepas[1,2], Yao Wang[1,2], Panagiotis Mistriotis[1,2,6], Selma A. Serra[7], John D. Lewis[5], Miguel A. Valverde[7], Stuart S. Martin[4,8], Sean X. Sun[1,2,9,10*], and Konstantinos Konstantopoulos[1,2,10,11*]

[1]Department of Chemical and Biomolecular Engineering, The Johns Hopkins University, Baltimore MD, 21218, USA

[2]Johns Hopkins Institute for NanoBioTechnology, The Johns Hopkins University, Baltimore MD, 21218, USA

[3]Department of Biomedical Engineering, Binghamton University, SUNY, Binghamton, NY, 13902, USA

[4] Marlene and Stewart Greenebaum National Cancer Institute Comprehensive Cancer Center, University of Maryland School of Medicine, Baltimore, MD, 21201, USA.

[5] Department of Oncology, University of Alberta, Edmonton, AB T6G 2E1, Canada

[6] Department of Chemical Engineering, Auburn University, Auburn, AL, 36849, USA.

[7]Laboratory of Molecular Physiology, Department of Experimental and Health Sciences, Universitat Pompeu Fabra, 08003 Barcelona, Spain

[8] Department of Physiology, University of Maryland School of Medicine, Baltimore, MD, 21201, USA.

[9] Department of Mechanical Engineering, The Johns Hopkins University, Baltimore MD, 21218, USA

[10] Department of Biomedical Engineering, The Johns Hopkins University, Baltimore MD, 21218, USA

[11] Department of Oncology, The Johns Hopkins University, Baltimore MD, 21205, USA

* Correspondence: konstant@jhu.edu (K.K.), ssun@jhu.edu (S.X.S.)

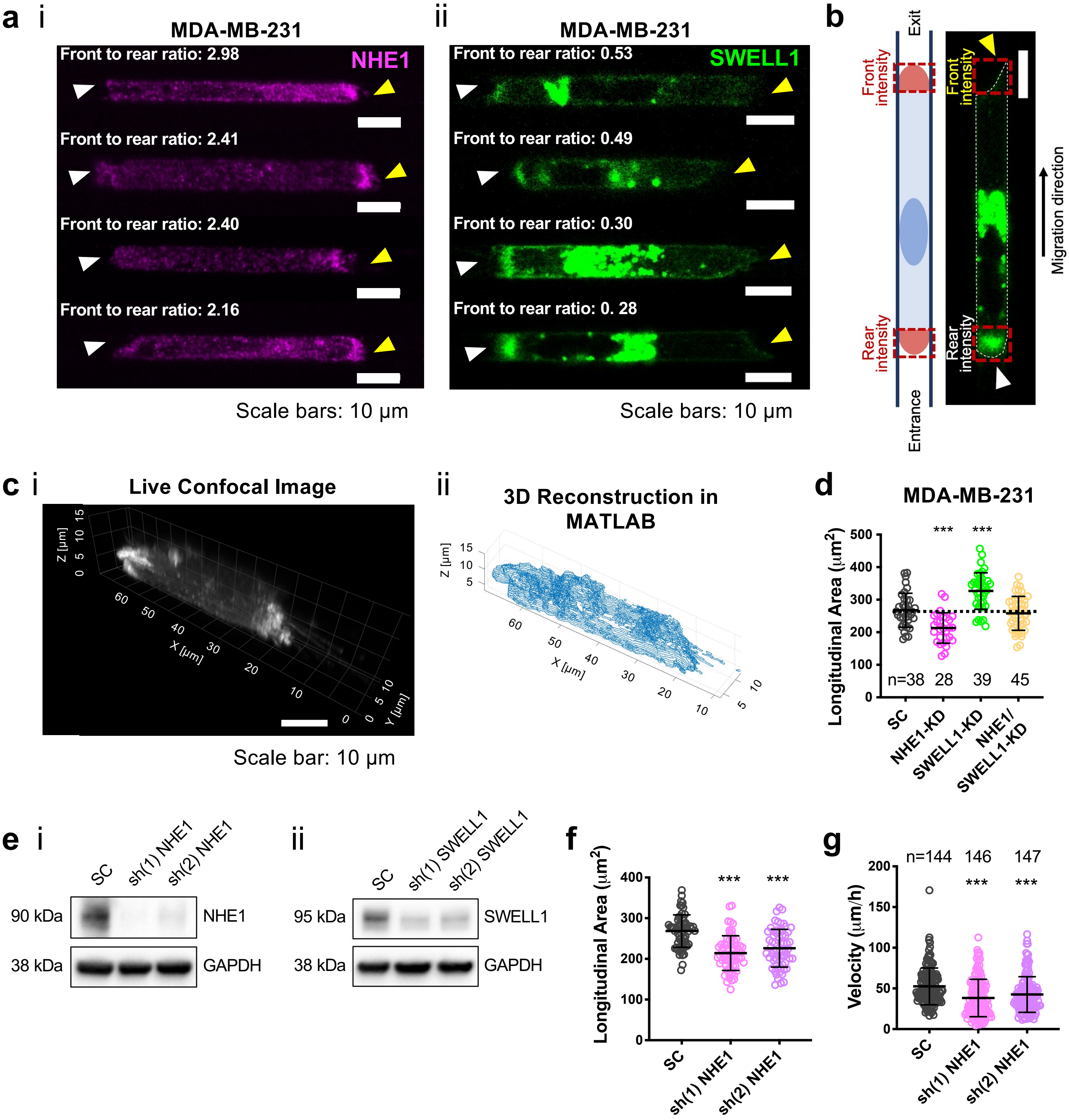

Supplementary Fig. 1. The spatial localization of NHE1 and SWELL1, and their roles in cell volume regulation and confined migration. **a** Representative confocal images of different MDA-MB-231 cells showing the preferential enrichment of (i) NHE1 and (ii) SWELL1 at the cell leading (yellow arrowheads) and trailing (white arrowheads) edges, respectively. The front to rear ratio of (i) NHE1 or (ii) SWELL1 intensity for each cell is shown. **b** Schematic (*left*) illustrating the cell region, denoted by red-dashed rectangles, that was segmented by a custom MATLAB script for intensity quantification of the front and rear of the confined cell (*right*) shown in (a,ii). **c** Representative (i) confocal image and (ii) 3D

reconstruction using MATLAB of an MDA-MB-231 cell inside a microchannel used for cell volume quantification. **d** Effects of NHE1 and/or SWELL1 knockdown on the longitudinal area of MDA-MB-231 cells inside confining channels. Data represent the mean ± SD for the indicated number of cells from 3 independent experiments. ***p<0.001 relative to SC and dual NHE1- and SWELL1-KD cells assessed by one-way ANOVA followed by Tukey's post-hoc test. **e** Representative western blots of MDA-MB-231 cells transduced with a non-targeting shRNA sequence (SC) or two different shRNA sequences against (i) NHE1 or (ii) SWELL1 from 3 independent experiments. GAPDH was used as loading control. **f, g** Effects of different shRNA sequences against NHE1 on (**f**) the longitudinal area and (**g**) migration velocity of MDA-MB-231 cells inside confining channels. Data represent the mean ± SD for (**f**) n=64 cells or (**g**) the indicated number of cells from 3 independent experiments. ***p<0.001 relative to SC assessed (**f**) by one-way ANOVA followed by Tukey's post-hoc test or (**g**) Kruskal-Wallis followed by Dunn's multiple comparisons test.

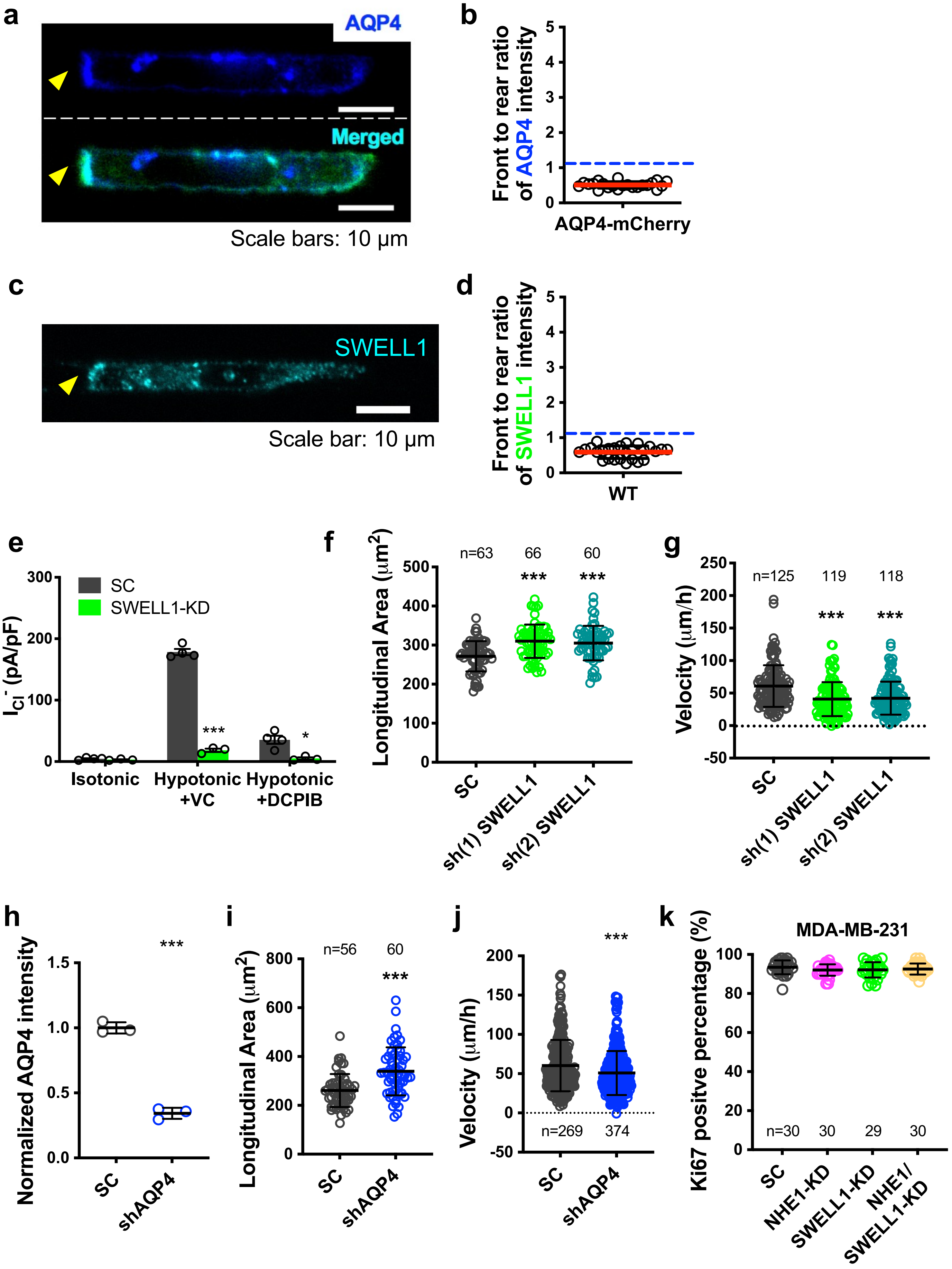
a
AQP4
Merged
Scale bars: 10 μm
b
Front to rear ratio of AQP4 intensity
AQP4-mCherry
c
SWELL1
Scale bar: 10 μm
d
Front to rear ratio of SWELL1 intensity
WT
e
SC
SWELL1-KD
I_Cl^- (pA/pF)
***
*
Isotonic
Hypotonic +VC
Hypotonic +DCPIB
f
Longitudinal Area (μm²)
n=63
66
60
***
***
SC
sh(1) SWELL1
sh(2) SWELL1
g
Velocity (μm/h)
n=125
119
118
***
***
SC
sh(1) SWELL1
sh(2) SWELL1
h
Normalized AQP4 intensity
***
SC
shAQP4
i
Longitudinal Area (μm²)
n=56
60
***
SC
shAQP4
j
Velocity (μm/h)
***
n=269
374
SC
shAQP4
k
MDA-MB-231
Ki67 positive percentage (%)
n=30
30
29
30
SC
NHE1-KD
SWELL1-KD
NHE1/SWELL1-KD

Supplementary Fig. 2. The spatial localization of SWELL1 and AQP4, and their roles in cell volume regulation and confined migration. **a** Colocalization of AQP4-mCherry (blue image, top) and SWELL1-GFP (green) for the same MDA-MB-231 cell shown in Fig. 1a. The merged image is shown at the bottom. Note their intense colocalization at the cell rear (arrowhead). **b** Front to rear ratio of AQP4 intensity in confined MDA-MB-231 cells. Data represent the mean ± SD for n=22 cells from 4 independent experiments. **c** Representative image of a confined MDA-MB-231 cell stained with a SWELL1 antibody showing preferential enrichment at the trailing edge (arrowhead). **d** Front to rear ratio of endogenous SWELL1 localization in confined MDA-MB-231 cells. Data represent the mean ± SD for n=26 cells from 2 independent experiments. **e** Whole-cell patch-clamp recordings of swelling activating currents in response to hypotonicity measured at +100 mV in SC and SWELL1-KD cells in the absence or presence of SWELL1 inhibitor, DCPIB (37.5 μM). Data represent mean ± SEM for n=4 (SC) or n=3 (SWELL1-KD) cells from 3 independent experiments *p<0.05, ***p<0.001 by two-tailed unpaired t-test. **f**, **g** Effects of different shRNA sequences against SWELL1 on (**f**) the longitudinal area and (**g**) migration velocity of MDA-MB-231 cells inside confining channels. Data represent the mean ± SD for the indicated number of cells from 3 independent experiments ***p<0.001 relative to SC assessed by (**f**) one-way ANOVA followed by Tukey's post-hoc test or (**g**) Kruskal-Wallis followed by Dunn's multiple comparisons test. **h** Fluorescence intensity of endogenous AQP4 in AQP4-KD relative to SC MDA-MB-231 cells plated on collagen I-coated 2D surfaces. Data are mean ± SD from 3 independent experiments. ***p<0.001 assessed by two-tailed unpaired t-test. **i, j** Effects of AQP4 knockdown on (**i**) the longitudinal area and (**j**) migration velocity of MDA-MB-231 inside confining channels. Data represent the mean ± SD for the indicated number of cells from 3 independent experiments. ***p<0.001 assessed by (**i**) two-tailed unpaired t-test or (**j**) two-tailed Mann-Whitney test. **k** Effects of NHE1 and/or SWELL1 knockdown on the percentage of Ki-67-positive MDA-MB-231 cells. Each dot represents the percentage of Ki-67-positive cells in each frame obtained from 3 independent experiments. Data are mean ± SD from the total number of frames indicated.

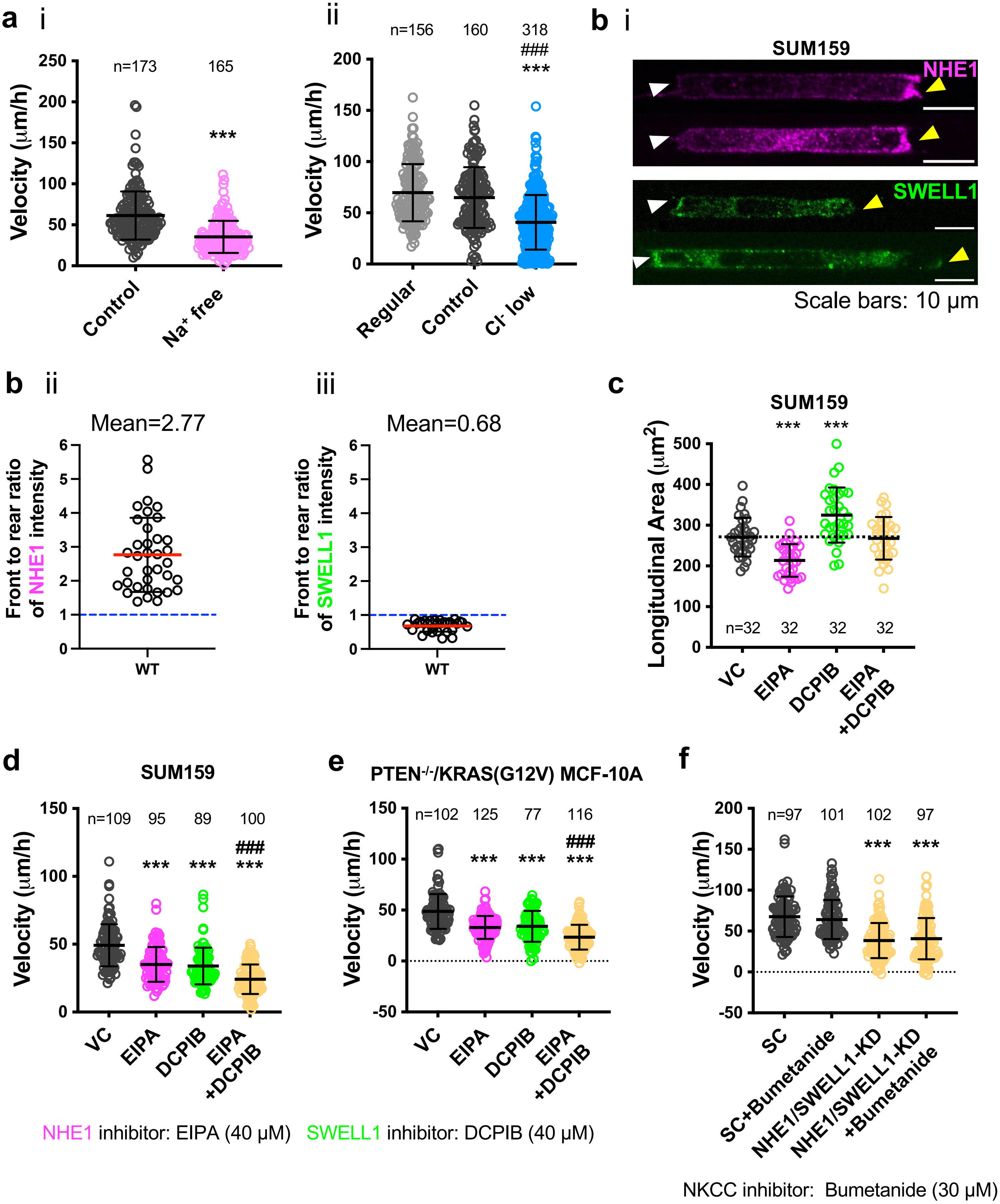
a i
Velocity (μm/h)
n=173
165
***
Control
Na+ free
ii
n=156
160
318
###
***
Regular
Control
Cl- low
b i
SUM159
NHE1
SWELL1
Scale bars: 10 μm
b ii
Mean=2.77
Front to rear ratio of NHE1 intensity
WT
iii
Mean=0.68
Front to rear ratio of SWELL1 intensity
WT
c
SUM159
Longitudinal Area (μm2)
n=32
32
32
32
VC
EIPA
DCPIB
EIPA +DCPIB
d
SUM159
n=109
95
89
100
e
PTEN-/-/KRAS(G12V) MCF-10A
n=102
125
77
116
f
n=97
101
102
97
SC
SC+Bumetanide
NHE1/SWELL1-KD
NHE1/SWELL1-KD +Bumetanide
NHE1 inhibitor: EIPA (40 μM)
SWELL1 inhibitor: DCPIB (40 μM)
NKCC inhibitor: Bumetanide (30 μM)

Supplementary Fig. 3. The spatial localization of NHE1 and SWELL1, and their roles in cell volume regulation and confined migration of different breast cancer cell lines. a Migration velocity of MDA-MB-231 cells in confining channels in the presence of (i) control or Na^+-free isotonic solutions, or (ii) regular, custom-made control or Cl^--low medium. Data represent the mean ± SD for the indicated number of cells from 4 independent experiments. ***p<0.001 relative to control medium, ###p<0.001 relative to regular medium, as assessed by (**a**i) two-tailed Mann-Whitney test or (**a**ii) one-way ANOVA followed by Tukey's post-hoc test. **b** Representative (i) confocal images of SUM159 cells showing preferential enrichment of NHE1 and SWELL1 at the cell leading (yellow arrowheads) and trailing edges (white arrowheads), respectively. Front to rear ratio of (ii) endogenous NHE1 or (iii) SWELL1 intensity in confined SUM159 cells. Data represent the mean ± SD for n=36 (bii) or n=30 (biii) cells from 3 independent experiments. **c-e** Effects of inhibiting NHE1 and/or SWELL1 on (**c**) the longitudinal area and confined migration of (**d**) SUM159 or (**e**) $PTEN^{-/-}$/KRAS(G12V) MCF-10A cells. EIPA (40 μM) and DCPIB (40 μM) inhibit NHE1 and SWELL1 function/activity, respectively. Data represent the mean ± SD for the indicated number of cells from 3 independent experiments. ***p<0.001 relative to VC. ###, p<0.001 relative to either of the single pharmacological inhibitors. Significance was determined using (**c**) one-way ANOVA followed by Tukey's post-hoc test or (**d**, **e**) Kruskal-Wallis followed by Dunn's multiple comparisons test. **f** Effect of inhibiting NKCC via Bumetanide (30 μM) on confined migration of either SC or dual NHE1 and SWELL1-KD MDA-MB-231 cells. Data represent the mean ± SD for the indicated number of cells from two independent experiments. ***p<0.001 relative to vehicle control SC cells, assessed by Kruskal-Wallis followed by Dunn's multiple comparisons test.

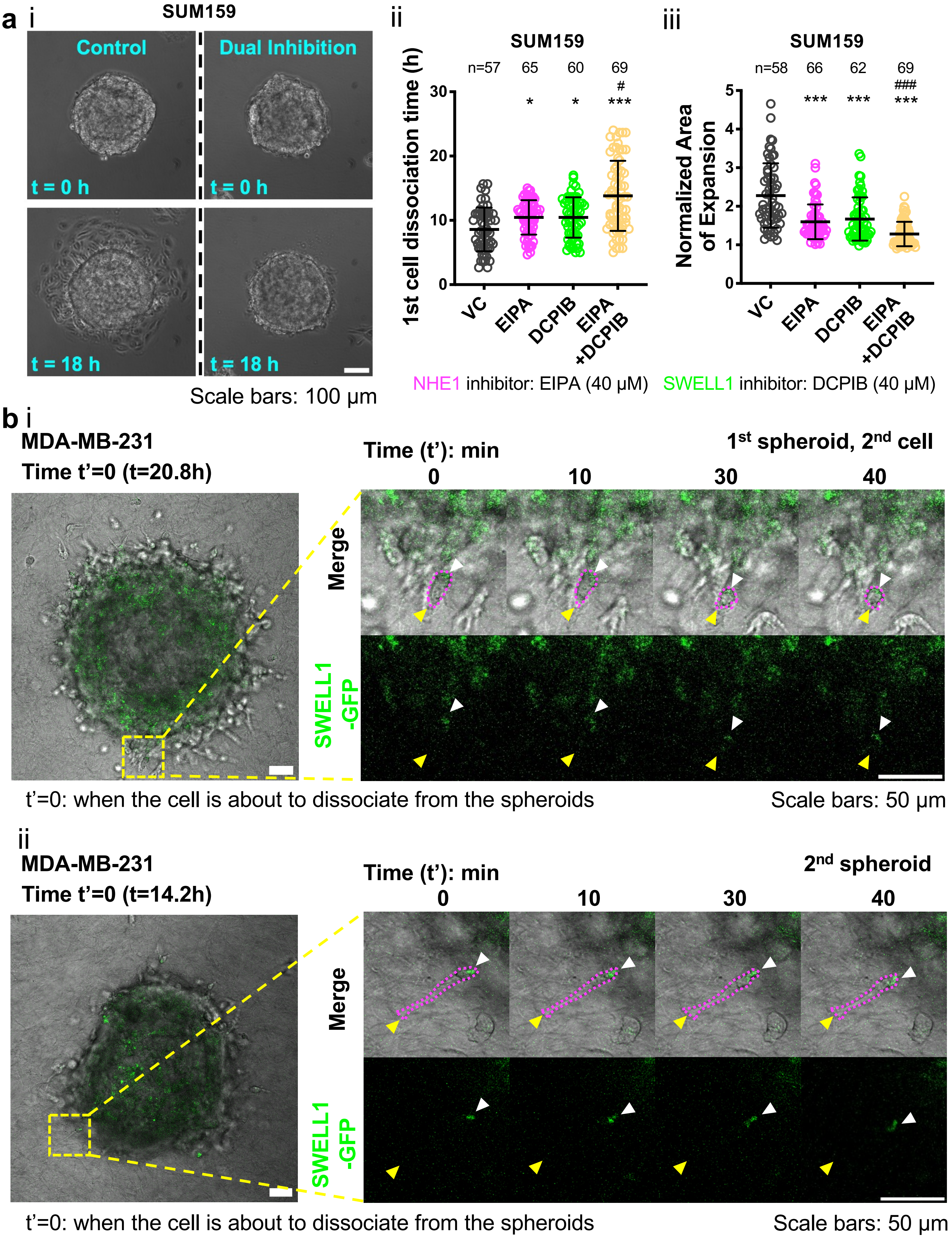
a i
SUM159
Control
Dual Inhibition
t = 0 h
t = 0 h
t = 18 h
t = 18 h
Scale bars: 100 µm
ii
SUM159
n=57 65 60 69
* * # ***
1st cell dissociation time (h)
0 10 20 30
VC EIPA DCPIB EIPA +DCPIB
iii
SUM159
n=58 66 62 69
*** *** ### ***
Normalized Area of Expansion
0 1 2 3 4 5
VC EIPA DCPIB EIPA +DCPIB
NHE1 inhibitor: EIPA (40 µM)
SWELL1 inhibitor: DCPIB (40 µM)
b i
MDA-MB-231
Time t'=0 (t=20.8h)
Time (t'): min
1st spheroid, 2nd cell
0 10 30 40
Merge
SWELL1 -GFP
t'=0: when the cell is about to dissociate from the spheroids
Scale bars: 50 µm
ii
MDA-MB-231
Time t'=0 (t=14.2h)
Time (t'): min
2nd spheroid
0 10 30 40
Merge
SWELL1 -GFP
t'=0: when the cell is about to dissociate from the spheroids
Scale bars: 50 µm

Supplementary Fig. 4. The roles of NHE1 and SWELL1 in cell dissociation from 3D breast cancer spheroids, and the polarization pattern of SWELL1 in this process. a Representative images showing (i) the effect of NHE1 and/or SWELL1 inhibition on SUM159 cell dissemination from spheroids embedded in 3D collagen gels at t=0 and 18 h. Effects of NHE1 and/or SWELL1 inhibition on (ii) the time for the first cell to dissociate from SUM159 spheroids and (iii) normalized area of expansion at t=18 h relative to t=0 embedded in 3D collagen gels. Data represent the mean ± SD for the indicated number of cells from 3 independent experiments. *p<0.05 and ***p<0.001 relative to VC, #p<0.05 and ###p<0.001 relative to either of single inhibited cells. Significance was determined using Kruskal-Wallis followed by Dunn's multiple comparisons test. **b** Time-lapse montage of representative SWELL1-GFP-tagged MDA-MB-231 cell (outlined by dashed magenta lines) dissociating from (i) the same spheroid as in Fig. 1n or (ii) a different spheroid embedded in a 3D collagen gel. SWELL1 polarization at the cell trailing edge is denoted by white arrowheads. The cell leading edge is indicated by yellow arrowheads. The lower part of the inset depicts SWELL1-GFP acquired by fluorescence confocal microscopy, whereas the upper part represents the merged image of DIC and GFP channels.

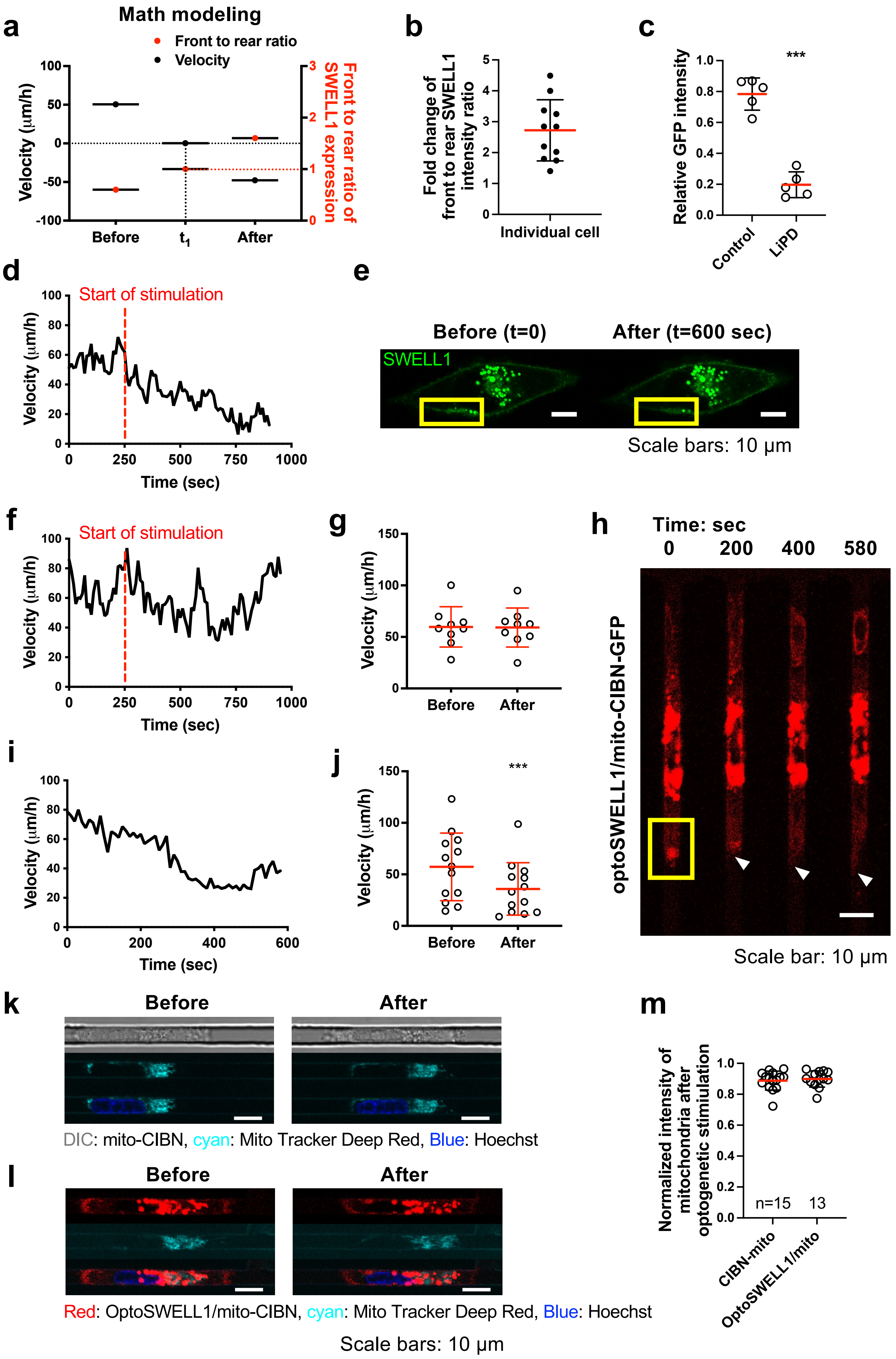
a
Math modeling
Front to rear ratio
Velocity
Velocity (μm/h)
Front to rear ratio of SWELL1 expression
Before
t1
After
b
Fold change of front to rear SWELL1 intensity ratio
Individual cell
c
Relative GFP intensity
***
Control
LiPD
d
Start of stimulation
Velocity (μm/h)
Time (sec)
e
Before (t=0)
After (t=600 sec)
SWELL1
Scale bars: 10 μm
f
Start of stimulation
Velocity (μm/h)
Time (sec)
g
Velocity (μm/h)
Before
After
h
Time: sec
0
200
400
580
optoSWELL1/mito-CIBN-GFP
Scale bar: 10 μm
i
Velocity (μm/h)
Time (sec)
j
***
Velocity (μm/h)
Before
After
k
Before
After
DIC: mito-CIBN, cyan: Mito Tracker Deep Red, Blue: Hoechst
l
Before
After
Red: OptoSWELL1/mito-CIBN, cyan: Mito Tracker Deep Red, Blue: Hoechst
Scale bars: 10 μm
m
Normalized intensity of mitochondria after optogenetic stimulation
n=15
13
CIBN-mito
OptoSWELL1/mito

Supplementary Fig. 5. Model prediction and control experiments for OptoSWELL1/mito-CIBN and LiPD system. **a** Model prediction of cell migration velocity when the front to rear ratio of SWELL1 intensity is 0.6, 1 or 1.6. **b** Relative fold change of front to rear SWELL1 signal intensity ratio following optogenetic stimulation of the 11 cells from 3 independent experiments shown in Fig. 2d. Data represent mean ± SD of the ratio of post- to pre- optogenetic stimulation. **c** SWELL1-GFP intensity after light stimulation normalized to pre-stimulation levels in MDA-MB-231 cells expressing either SWELL1-GFP only or both SWELL1-GFP and LiPD system. Data represent the mean ± SD for 5 cells from 2 independent experiments. ***$p<0.001$ by two-tailed paired t-test. **d** Instantaneous migration velocity of the cell shown in Fig. 2f. **e** Representative images of an MDA-MB-231 cell expressing SWELL1-GFP only before (t=0) and after 600 sec of stimulation with blue light (488 nm) in the region enclosed by the yellow box (see quantification in **c** above). **f** Instantaneous migration velocity of an MDA-MB-231 cell expressing SWELL1-GFP only before and after stimulation with blue light (488 nm). **g** Migration velocity of MDA-MB-231 cells expressing SWELL1-GFP only before and after light stimulation. Data represent the mean ± SD for 9 cells from 4 independent experiments. **h** Time-lapse montage of a representative MDA-MB-231 cell expressing OptoSWELL1 and mito-CIBN-GFP following light stimulation at the cell trailing edge (yellow box). Gradual reduction of SWELL1 intensity at the cell rear (white arrowhead) is observed with time. **i** Instantaneous migration velocity of an MDA-MB-231 cell expressing OptoSWELL1 and mito-CIBNGFP after light-induced downregulation of SWELL1 at the cell rear. **j** Migration velocity of MDA-MB-231 cells expressing OptoSWELL1 and mito-CIBN-GFP before and after stimulation. Data represent the mean ± SD for 13 cells from 3 independent experiments. ***$p<0.001$ by two-tailed paired t-test. **k** Representative images of an MDA-MB-231 cell (DIC, *top*) expressing only mito-CIBN-GFP, Mito Tracker Deep Red (cyan, *middle*) and Hoechst (blue merged with cyan, *bottom*) before and after light stimulation at the cell trailing edge. **l** Representative images of an MDA-MB-231 cell expressing OptoSWELL1 (red, *top*), mito-CIBN-GFP and Mito Tracker Deep Red (cyan, *middle*) and Hoechst (blue merged with red and cyan, *bottom*) before and after optogenetic downregulation of SWELL1 at the cell trailing edge. **m** Fluorescence intensity of mitochondria detected by Mito Tracker Deep Red after light stimulation normalized to pre-stimulation levels in MDA-MB-231 expressing either Mito-CIBN-GFP or OptoSWELL1 and Mito-CIBN-GFP. Data represent mean ± SD for the indicated number of cells from 3 independent experiments.

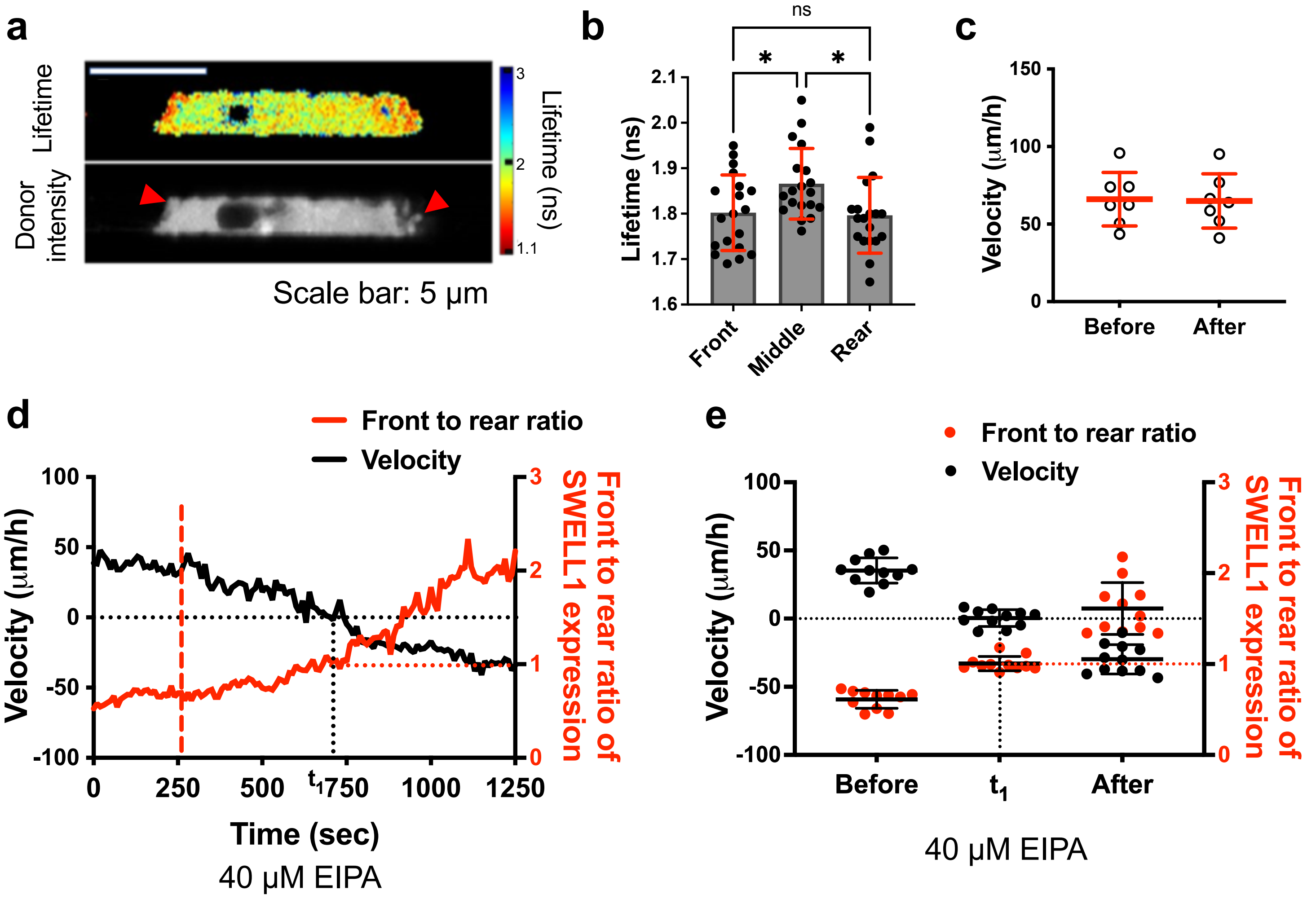

Supplementary Fig. 6. RhoA activity regulates SWELL1 polarization. **a** Heat map (*top*) of donor fluorescence lifetime (nsec) using a RhoA activity biosensor in a cell migrating inside confining channels as imaged by FLIM/FRET. Arrowheads point to membrane blebs. **b** Spatial distribution of RhoA activity at different segments of cells migrating inside confining channels. Data are mean ± SD for n=19 cells from 2 independent experiments. *p<0.05. Significance was determined using one-way ANOVA followed by Tukey's post-hoc test. **c** Migration velocity of MDA-MB-231 cells expressing OptoGEF-RhoA and SWELL1-iRFP, but not mito-CIBN-GFP, before and after light stimulation. Data represent the mean ± SD for 7 cells from 4 independent experiments. p=0.79 by two-tailed paired t-test. **d** Instantaneous migration velocity (black line) and front to rear ratio of SWELL1 intensity (red line) of an MDA-MB-231 cell expressing OptoSWELL1 and CAAX-CIBN-GFP treated with 40 μM EIPA. **e** Migration velocity (black dots) and front to rear ratio of SWELL1 intensity (red) of MDA-MB-231 cells expressing OptoSWELL1 and CAAX-CIBN-GFP that were treated with 40 μM EIPA before optogenetic stimulation, at $t=t_1$ and $t \geq 1{,}000$ sec after stimulation. Data represent the mean ± SD for 11 cells from 3 independent experiments.

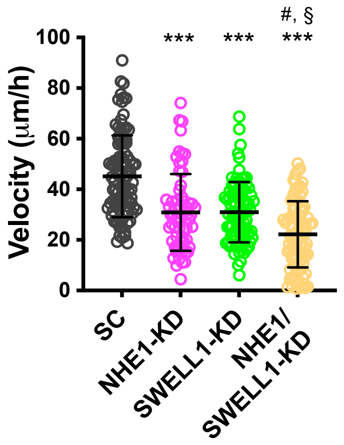

Supplementary Fig. 7. Effects of NHE1 and/or SWELL1 knockdown on the migration velocity of luciferase-labeled MDA-MB-231 cells. Data represent mean ± SD for n=98 (SC), n=65 (NHE1-KD), n=87 (SWELL1-KD) and n=91 (dual KD) cells from 3 independent experiments. ***p<0.001 relative to SC, #p=0.0175 relative to NHE1-KD cells, §p=0.0011 relative to SWELL1-KD cells, assessed by Kruskal-Wallis followed by Dunn's multiple comparisons test.

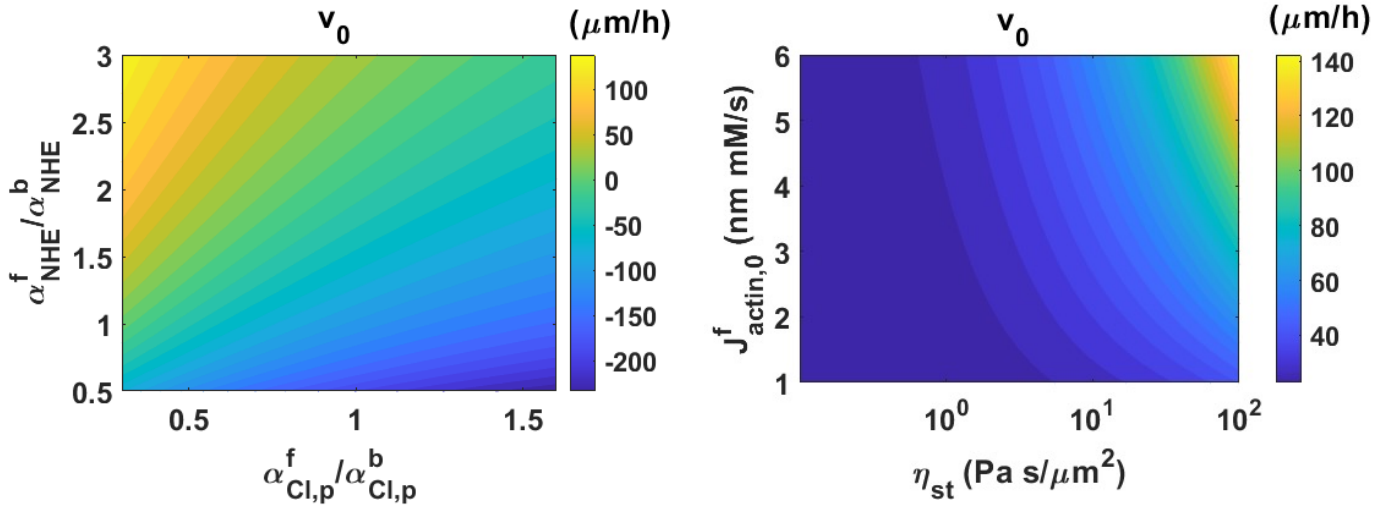

Supplementary Fig. 8. Model predictions on the dependence of cell velocity on different parameters. *Left panel*: contour of cell velocity as a function of NHE1 and SWELL1 polarization ratios. *Right panel*: contour of cell velocity as a function of the rate of actin polymerization and the strength of focal adhesions. Color bars are cell velocities in μm/h.

Supplementary Table 1: Table showing single- and dual- knockdown cell lines used in *in vitro* and *in vivo* experiments.

***In vitro* experiments**

SC	NHE1-KD	SWELL1-KD	NHE1/SWELL1-KD
pLVTHM-SC<br>pLKO.1-SC	pLVTHM-sh1&2NHE1<br>pLKO.1-SC	pLVTHM-SC<br>pLKO.1-sh1SWELL1	pLVTHM-sh1&2NHE1<br>pLKO.1-sh1SWELL1

***In vivo* experiments**

SC	NHE1-KD	SWELL1-KD	NHE1/SWELL1-KD
pLKO.1-SC	pLKO.1-sh1&2NHE1	pLKO.1-sh1SWELL1	pLKO.1-sh1&2NHE1<br>pLKO.1-sh1SWELL1

Supplementary Table 2: Table showing parameters and their values used in the model.

Parameter	Description	Value	Source
T (K)	Absolute temperature	310	Physiological condition
L (μm)	Cell length	50	Experiment condition
b (μm)	Cell width	3	Experiment condition
w (μm)	Cell depth	10	Experiment condition
η (Pa·s/μm ^{2}/mM)	Drag coefficient between two phases	10^{-3}	Based on Ref[1].
η_{st} (Pa·s/μm ^{2}/mM)	Coefficient of drag from focal adhesion	40	Fitted
k_{σ_n} (Pa/mM)	Coefficient of the passive F-actin stress	1×10^3	Estimated
ξ (Pa·s/μm)	Friction Coefficient	50	Fitted
d_g (Pa·s/μm)	Coefficient of hydraulic pressure	30	Fitted
D_{θ_c} (μm ^{2}/s)	Diffusion coefficient of G-actin	10	Ref[2].
θ_* (mM)	Average concentration of total actin	0.3	Based on Ref[3,4].
J_a (nm mM/s)	Coefficient in $J_{\mathrm{actin}}^{\mathrm{f}} = J_a \theta_c^{\mathrm{f}} / (\theta_{c,c} + \theta_c^{\mathrm{f}})$	2.2	Fitted
$\theta_{c,c}$ (μM)	Critical value of actin polymerization	0.2	Ref[4].
γ (1/s)	Rate of actin depolymerization	0.5	Estimated
N_A (pmol)	Total intracellular A^-	9.5×10^{-2}	Estimated
$N_{\mathrm{Buf}} + N_{\mathrm{HBuf}}$ (pmol)	Total intracellular Buffer Solution	9.5×10^{-2}	Estimated
α_{w} (m/Pa/s)	Permeability coefficient of water	10^{-10}	Ref[5].
$\alpha_{\mathrm{Na},p}^{\mathrm{f}(b)}$ (mol ^{2}/J/μm ^{2}/s)	Permeability of Na at front (back)	0.1	Estimated
$\alpha_{\mathrm{K},p}^{\mathrm{f}(b)}$ (mol ^{2}/J/μm ^{2}/s)	Permeability of K at front (back)	20	Estimated
$\alpha_{\mathrm{Cl},p}^{\mathrm{f}}$ (mol ^{2}/J/μm ^{2}/s)	Permeability of Cl at front	70	Estimated
$\alpha_{\mathrm{Cl},p}^{\mathrm{b}}$ (mol ^{2}/J/μm ^{2}/s)	Permeability of Cl at back	$\alpha_{\mathrm{Cl},p}^{\mathrm{f}}/0.6$	From experiment
$\alpha_{\mathrm{NKE}}^{\mathrm{f}(b)}$ (mol/μm ^{2}/s)	Permeability of NKE at front (back)	3.9×10^3	Estimated
$\alpha_{\mathrm{NHE}}^{\mathrm{f}}$ (mol ^{2}/J/μm ^{2}/s)	Permeability of NHE at front	500	Estimated
$\alpha_{\mathrm{NHE}}^{\mathrm{b}}$ (mol ^{2}/J/μm ^{2}/s)	Permeability of NHE at back	$\alpha_{\mathrm{NHE}}^{\mathrm{f}}/2.3$	From experiment
$\alpha_{\mathrm{AE2}}^{\mathrm{f}}$ (mol ^{2}/J/μm ^{2}/s)	Permeability of AE2 at front	100	Estimated
$\alpha_{\mathrm{AE2}}^{\mathrm{b}}$ (mol ^{2}/J/μm ^{2}/s)	Permeability of AE2 at back	280	Fitted
$\beta_{\mathrm{NKE,Na}}$	Constant in J_{NKE}	0.1	Estimated
$\beta_{\mathrm{NKE,K}}$	Constant in J_{NKE}	0.01	Estimated
β_1 (m/N)	Constant in G_m	2×10^3	Estimated
β_2 (N/m)	Constant in G_m	5×10^{-4}	Estimated
β_3 (1/mV)	Constant in $G_{V,\mathrm{NKE}}$	0.03	Ref[6].
β_4 (mV)	Constant in $G_{V,\mathrm{NKE}}$	-150	Ref[6].
β_5	Constant in G_{NHE}	15	Ref[7].
β_6	Constant in G_{NHE}	7.2	Ref[7].
β_7	Constant in G_{AE2}	10	Ref[7].
β_8	Constant in G_{AE2}	7.1	Ref[7].
k_H (atm/M)	Henry's constant	29	Ref[8].
$P_{\mathrm{CO_2}}$ (atm)	Partial pressure of CO_2	5%	Physiological condition

pK_c	pK for bicarbonate-carbonic acid pair	6.1	Ref[8].
pK_B	pK for intracellular buffer	7.5	Based on Ref[8].
$c_{\mathrm{N}a}^0$ (mM)	Na^+ concentration in the medium	145	Physiological condition
c_{K}^0 (mM)	K^+ concentration in the medium	9	Physiological condition
c_{Cl}^0 (mM)	Cl^- concentration in the medium	105	Physiological condition
$c_{\mathrm{HCO_3}}^0$ (mM)	HCO_3^- concentration in the medium	35	Physiological condition
c_{G}^0 (mM)	Glucose concentration in the medium	25	Physiological condition

Supplementary References

1 Dembo, M. & Harlow, F. Cell motion, contractile networks, and the physics of interpenetrating reactive flow. *Biophys J* **50**, 109-121 (1986).

2 Zicha, D. *et al.* Rapid actin transport during cell protrusion. *Science* **300**, 142-145 (2003).

3 Satcher, R. L. & Dewey, C. F. Theoretical estimates of mechanical properties of the endothelial cell cytoskeleton. *Biophys J* **71**, 109-118 (1996).

4 Pollard, T. D., Blanchoin, L. & Mullins, R. D. Molecular mechanisms controlling actin filament dynamics in nonmuscle cells. *Annu Rev Biophys Biomol Struct* **29**, 545-576 (2000).

5 Jiang, H. & Sun, S. X. Cellular pressure and volume regulation and implications for cell mechanics. *Biophys J* **105**, 609-619 (2013).

6 Gadsby, D. C., Kimura, J. & Noma, A. Voltage dependence of Na/K pump current in isolated heart cells. *Nature* **315**, 63-65 (1985).

7 Casey, J. R., Grinstein, S. & Orlowski, J. Sensors and regulators of intracellular pH. *Nat Rev Mol Cell Biol* **11**, 50-61 (2010).

8 Weinstein, A. M. A mathematical model of the rat proximal tubule. *Am J Physiol* **250**, F860-873 (1986).
