# Supplementary material for: Polarized NHE1 and SWELL1 regulate migration direction, efficiency and metastasis
Source: Nat Commun. 2022 Oct 17;13:6128. doi: 10.1038/s41467-022-33683-1 (PMC9576788; doi:10.1038/s41467-022-33683-1)
Supplement: Supplementary file 3 — Description of additional Supplementary File [file 41467_2022_33683_MOESM3_ESM.pdf]

Descriptions of Additional Supplementary Data files

Supplementary Movie S1. Live-cell confocal microscopy showing preferential enrichment of SWELL1-GFP at the trailing edge of an MDA-MB-231 cell migrating in confinement. Scale bar: 10 µm.

Supplementary Movie S2. Live-cell confocal microscopy showing preferential enrichment of AQP4-mCherry at the trailing edge of an MDA-MB-231 cell migrating in confinement. Scale bar: 10 µm.

Supplementary Movie S3. Time-lapse microscopy showing dissemination of scramble control (SC) and dual NHE1/SWELL1-KD MDA-MB-231 cells from spheroids embedded into 3D collagen gels. Scale bar: 50 µm.

Supplementary Movie S4. Time-lapse microscopy showing dissemination of scramble control (SC) and dual NHE1/SWELL1-KD MDA-MB-231 cells from spheroids placed on 2D collagen I-coated surfaces. Scale bar: 50 µm.

Supplementary Movie S5. Migration of an MDA-MB-231 cell expressing OptoSWELL1 and CAAX-CIBN-GFP inside a confining channel following light stimulation at the cell leading edge during the entire time period as described in the experimental procedures. The yellow box outlines the initial area of light stimulation. Scale bar: 10 µm.

Supplementary Movie S6. Migration of an MDA-MB-231 cell expressing OptoGEF, CAAX-CIBN-GFP and SWELL1-iRFP inside a 10 µm-wide channel following light stimulation at the cell leading edge during the entire time period as described in the experimental procedures. The yellow box outlines the initial area of light stimulation. Scale bar: 10 µm.

Supplementary Movie S7. Migration of an MDA-MB-231 cell expressing OptoGEF, CAAX-CIBN-GFP and SWELL1-KD inside a 10 µm-wide channel following light stimulation at the cell leading edge after 4 min as described in the experimental procedures. The yellow box outlines the initial area of light stimulation. Scale bar: 10 µm.
