# Supplementary material for: Polarized NHE1 and SWELL1 regulate migration direction, efficiency and metastasis
Source: Nat Commun. 2022 Oct 17;13:6128. doi: 10.1038/s41467-022-33683-1 (PMC9576788; doi:10.1038/s41467-022-33683-1)
Supplement: Supplementary file 11 — Supplementary Software [file 41467_2022_33683_MOESM11_ESM.zip › Supplementary Software Information/README.docx]

1. System requirements
   - Software dependencies: MATLAB (Version R2016b), Microsoft Excel (Version 15.30), ImageJ (Version 2.0.0-rc-56/1.51h)
   - Operating system: macOS Sierra (Version 10.12.6)
   - Versions the software has been tested on: MATLAB (Version R2016b), Microsoft Excel (Version 15.30), ImageJ (Version 2.0.0-rc-56/1.51h)
   - Hardware requirements: only standard computer is required with enough RAM to support MATLAB operations
2. Installation guide
   - Instructions to install MATLAB: <https://www.mathworks.com/help/install/>
   - Instructions to install Microsoft Excel: <https://support.microsoft.com/en-us/office/download-and-install-or-reinstall-microsoft-365-or-office-2021-on-a-pc-or-mac-4414eaaf-0478-48be-9c42-23adc4716658>
   - Instructions to install ImageJ: <https://imagej.nih.gov/ij/plugins/fraclac/FLHelp/Installation.htm>
   - Typical install time on a “normal” desktop computer: around 10 to 15 minutes, also depends on internet connection speed.
3. Analyzing the velocity and front-to-rear ratio of cells with optogenetic expression
   - Install MATLAB, Microsoft Excel and ImageJ
   - To analyze center position of the cell:
     - Open MATLAB script “Optogenetic_CAAX_OptoSWELL1_speed”
     - Change the FileName to “Demo_Optogenetics.tif”
     - Click Run
     - Copy the data of center_posi (in pixel) to Excel file as shown in “Demo_Output_OptoSWELL1.xlsx”
     - This step takes about 5 minutes on a “normal” desktop computer
   - To calculate cell velocity
     - Calculate the cell displacement (pixel) by using Excel file as shown in “Demo_Output_OptoSWELL1.xlsx”
     - Calculate the velocity by using Excel file as shown in “Demo_Output_OptoSWELL1.xlsx”. Micron/pixel ratio can be found in image property in ImageJ. In this demo, the ratio is 0.1206.
     - Moving average of cell velocity is calculated using MATLAB: A = movmean(B,[25 25]). A: moving average of cell velocity B: cell velocity calculated by Excel file.
     - Copy the column of moving average to Excel file, as shown in “Demo_Output_OptoSWELL1.xlsx”
     - This step takes about 10 minutes on a “normal” desktop computer
   - To analyze front-to-rear ratio of the cell
     - Open MATLAB script “Optogenetic_CAAX_OptoSWELL1”
     - Change the FileName to “Demo_Optogenetics.tif”
     - Click Run
     - Use the cursor to select and click on the cell leading edge, then the cell trailing edge
     - Copy the data of front_to_rear_ratio to Excel file as shown in “Demo_Output_OptoSWELL1.xlsx”
     - This step takes about 10 minutes on a “normal” desktop computer

The code for the multi-phase model was written in MATLAB and can be run in standard MATLAB environment. This code will be provided upon request.
