# Supplementary figures and images for: Polarized NHE1 and SWELL1 regulate migration direction, efficiency and metastasis
Source: Nat Commun. 2022 Oct 17;13:6128. doi: 10.1038/s41467-022-33683-1 (PMC9576788; doi:10.1038/s41467-022-33683-1)

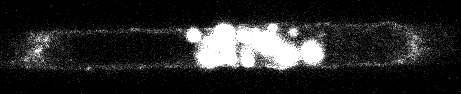

Supplement: Supplementary file 11 — Supplementary Software [file 41467_2022_33683_MOESM11_ESM.zip › Supplementary Software Information/Demo_Optogenetics.tif]
