# Supplementary material for: Polarized NHE1 and SWELL1 regulate migration direction, efficiency and metastasis
Source: Nat Commun. 2022 Oct 17;13:6128. doi: 10.1038/s41467-022-33683-1 (PMC9576788; doi:10.1038/s41467-022-33683-1)
Supplement: Supplementary file 12 — Reporting Summary [file 41467_2022_33683_MOESM12_ESM.pdf]

nature portfolio

Corresponding author(s): Konstantinos Konstantopoulos
Sean X. Sun

Last updated by author(s): Sep 8, 2022

Reporting Summary

Nature Portfolio wishes to improve the reproducibility of the work that we publish. This form provides structure for consistency and transparency in reporting. For further information on Nature Portfolio policies, see our Editorial Policies and the Editorial Policy Checklist.

Statistics

For all statistical analyses, confirm that the following items are present in the figure legend, table legend, main text, or Methods section.

n/a	Confirmed	
☐	☒	The exact sample size (*n*) for each experimental group/condition, given as a discrete number and unit of measurement
☐	☒	A statement on whether measurements were taken from distinct samples or whether the same sample was measured repeatedly
☐	☒	The statistical test(s) used AND whether they are one- or two-sided<br>*Only common tests should be described solely by name; describe more complex techniques in the Methods section.*
☐	☒	A description of all covariates tested
☐	☒	A description of any assumptions or corrections, such as tests of normality and adjustment for multiple comparisons
☐	☒	A full description of the statistical parameters including central tendency (e.g. means) or other basic estimates (e.g. regression coefficient) AND variation (e.g. standard deviation) or associated estimates of uncertainty (e.g. confidence intervals)
☐	☒	For null hypothesis testing, the test statistic (e.g. *F*, *t*, *r*) with confidence intervals, effect sizes, degrees of freedom and *P* value noted<br>*Give P values as exact values whenever suitable.*
☒	☐	For Bayesian analysis, information on the choice of priors and Markov chain Monte Carlo settings
☒	☐	For hierarchical and complex designs, identification of the appropriate level for tests and full reporting of outcomes
☒	☐	Estimates of effect sizes (e.g. Cohen's *d*, Pearson's *r*), indicating how they were calculated

Our web collection on statistics for biologists contains articles on many of the points above.

Software and code

Policy information about availability of computer code

Data collection	Data were collected by using standard features of NIS-Elements (Version 5.02.01 or 4.13.05), Axon p-Clamp (Version 10), ZEN 2.3 SP1 FP3 (black) or SymPhoTime 64 (Version 2.4).
Data analysis	Data were primarily collected and organized in Microsoft Excel (Version 15.30) and GraphPad Prism (Version 7.0b or 9.1.1). Select analyses were performed in ImageJ (Version 2.0.0/1.51h), MATLAB (Version R2016b), SymPhoTime 64 (Version 2.4) or Imaris (Version 9.7.0), as described in Methods. The code for the two-phase model is available on GitHub in a link provided by SXS: https://github.com/sxslabjhu/

For manuscripts utilizing custom algorithms or software that are central to the research but not yet described in published literature, software must be made available to editors and reviewers. We strongly encourage code deposition in a community repository (e.g. GitHub). See the Nature Portfolio guidelines for submitting code & software for further information.

Data

Policy information about availability of data

All manuscripts must include a data availability statement. This statement should provide the following information, where applicable:

- Accession codes, unique identifiers, or web links for publicly available datasets
- A description of any restrictions on data availability
- For clinical datasets or third party data, please ensure that the statement adheres to our policy

The main data supporting the results of this study are available within the Article and its Supplementary Information. All source data are provided with this paper. Patient distant metastasis free survival (DMFS) time and gene expression data are available through KM-plotter: https://kmplot.com/analysis/

Field-specific reporting

Please select the one below that is the best fit for your research. If you are not sure, read the appropriate sections before making your selection.

☒ Life sciences ☐ Behavioural & social sciences ☐ Ecological, evolutionary & environmental sciences

For a reference copy of the document with all sections, see nature.com/documents/nr-reporting-summary-flat.pdf

Life sciences study design

All studies must disclose on these points even when the disclosure is negative.

Sample size	Supplementary Information with the exact sample size, number of replicates and p value for each experiment are provided. No predetermination of sample size was done. Sample size was chosen based on the throughput of the technique used. Sample sizes were sufficient to show the same trends between the replicates performed for each experiment, and by statistical testing. For animal experiments, no statistical methods were used to calculate sample size and group size, and the sample size was determined based on experience of similar assays performed earlier. In each experiment multiple cells or animals were examined in parallel leading to sample sizes primarily of the order of tens to hundreds.
Data exclusions	For in vitro migration experiments, dividing or apoptotic cells were excluded from analysis based on pre-established criteria. For bioluminescence imaging of cancer cell metastasis in mice, one mouse injected with NHE1-KD cells died before imaging organs to quantify metastasis (n=9). In addition, one extreme outlier was excluded from the analysis (lymph nodes of a mouse injected with dual NHE1-/SWELL1-KD cells) because the value was found to be at least 40 times greater than any other value obtained from the lymph nodes of mice injected with SC, single or dual KD cells.
Replication	Most experiments were repeated 3 or more times, with similar results observed each time. Select control experiments were repeated 2 times with consistent data across all replicates. The exact number of replicates for each experiment has been indicated in the figure legends.
Randomization	Mice and chick embryos were assigned randomly to experimental groups. For all other experiments, cells were randomly distributed into experimental groups before imaging and analysis.
Blinding	The in vivo mouse experiments were performed in a blinded manner. For remaining experiments researchers were not blinded as data collection and analysis were performed by the same individual assigning the groups. Wherever possible findings (e.g., cell migration speeds, cell volume) were analyzed in an unbiased manner by the use of automated analysis codes in ImageJ and MATLAB.

Reporting for specific materials, systems and methods

We require information from authors about some types of materials, experimental systems and methods used in many studies. Here, indicate whether each material, system or method listed is relevant to your study. If you are not sure if a list item applies to your research, read the appropriate section before selecting a response.

Materials & experimental systems

n/a	Involved in the study	
☐	☒	Antibodies
☐	☒	Eukaryotic cell lines
☒	☐	Palaeontology and archaeology
☐	☒	Animals and other organisms
☒	☐	Human research participants
☒	☐	Clinical data
☒	☐	Dual use research of concern

Methods

n/a	Involved in the study	
☒	☐	ChIP-seq
☒	☐	Flow cytometry
☒	☐	MRI-based neuroimaging

Antibodies

Antibodies used

For immunofluorescence:
anti-NHE1 (1:50; mouse, clone 54, Santa Cruz Biotechnology, sc-136239)
anti-AQP4 (1:50; mouse, clone 4/18, Santa Cruz Biotechnology, sc-32739)
anti-Ki-67 (1:800; clone 8D5, 9449, Cell Signaling Technology)
SWELL1 (LRRC8A; 1:100) antibody is a kind gift from Thomas J. Jentsch
Alexa Fluor 488 goat anti-mouse immunoglobulin-G (IgG) (H+L) (1:100; A11029, lot # 2179204, Invitrogen)
Alexa Fluor 568 goat anti-rabbit IgG (H+L) (1:200; A11011, lot # 2277758, Invitrogen)
Alexa Fluor Plus 647 goat anti-mouse IgG (H+L) (1:100; A32728, lot # WE322197, Invitrogen)
Alexa Fluor Plus 647 goat anti-rabbit IgG (H+L) (1:100; A21245, lot # 2299231, Invitrogen)

For immunoblotting:
anti-NHE1 (1:200; mouse, clone 54, Santa Cruz Biotechnology, sc-136239)

anti-SWELL1 (1:100; mouse, clone 8H9, Santa Cruz Biotechnology, sc-517113)
GAPDH (1:1000; rabbit, clone 14C10, Cell Signaling Technology 2118)
anti-mouse IgG HRP-linked antibody (1:2000; Cell Signaling Technologies, 7076)
anti-rabbit IgG HRP-linked antibody (1:2000; Cell Signaling Technologies, 7074)

Validation

Antibodies for immunofluorescence were validated by the manufacturer prior to purchasing:
anti-NHE1: https://datasheets.scbt.com/sc-136239.pdf
anti- AQP4: https://datasheets.scbt.com/sc-32739.pdf
anti-Ki67: https://media.cellsignal.com/coa/9449/14/9449-lot-14-coa.pdf
Alexa Fluor 488 goat anti-mouse: https://www.thermofisher.com/document-connect/document-connect.html?url=https%3A%2F%2Fassets.thermofisher.com%2FTFS-Assets%2FBID%2Fcertificate%2FCertificates-of-Analysis%2FA11029%2520Lot%25202179204%2520CofA.pdf&title=TG90ICMmbmJzcDsyMTc5MjA0
Alexa Fluor 568 goat anti-rabbit: https://www.thermofisher.com/document-connect/document-connect.html?url=https%3A%2F%2Fassets.thermofisher.com%2FTFS-Assets%2FBID%2Fcertificate%2FCertificates-of-Analysis%2FA11011%2520Lot%25202277758%2520CofA.pdf&title=TG90ICMmbmJzcDsyMjc3NzU4
Alexa Fluor Plus 647 goat anti-mouse: https://www.thermofisher.com/document-connect/document-connect.html?url=https%3A%2F%2Fassets.thermofisher.com%2FTFS-Assets%2FLSG%2Fcertificate%2FCertificates-of-Analysis%2FA32728_WE322197.PDF&title=TG90ICMmbmJzcDtXRTMyMjE5Nw==
Alexa Fluor Plus 647 goat anti-rabbit: https://www.thermofisher.com/document-connect/document-connect.html?url=https%3A%2F%2Fassets.thermofisher.com%2FTFS-Assets%2FBID%2Fcertificate%2FCertificates-of-Analysis%2FA21245%2520Lot%25202299231%2520CofA.pdf&title=TG90ICMmbmJzcDsyMjk5MjMx

anti-SWELL1 antibody for immunofluorescence was verified based on the cellular distribution previously reported by Thomas J. Jentsch and others. Commercially available antibodies were also verified by comparing their cellular distribution to that provided in the manufacturer's datasheet.

Antibodies for immunoblotting were also validated by the manufacturer prior to purchasing:
anti-SWELL1: https://datasheets.scbt.com/sc-517113.pdf
anti-GAPDH: https://media.cellsignal.com/coa/2118/14/2118-lot-14-coa.pdf
anti-mouse IgG HRP-linked antibody: https://media.cellsignal.com/coa/7076/38/7076-lot-38-coa.pdf
anti-rabbit IgG HRP-linked antibody: https://media.cellsignal.com/coa/7074/31/7074-lot-31-coa.pdf

Antibodies for immunoblotting were also verified based on the appropriate molecular weight of the protein probed.

Eukaryotic cell lines

Policy information about cell lines

Cell line source(s)

The parental MDA-MB-231 cell line was purchased from American Type Culture Collection (ATCC). SUM159 cells were a gift from Denis Wirtz (Johns Hopkins University). PTEN-/-/KRAS(G12V) MCF-10A cells were a gift from Michele I. Vitolo (University of Maryland at Baltimore). Select cell lines were modified from the parental line, as described in Methods (e.g., development of cell lines with scramble, shRNA or live reporters).

Authentication

Cell lines were originally authenticated by ATCC, and were not further authenticated as part of this study.

Mycoplasma contamination

All cell lines are tested negative for mycoplasma contamination, as assessed by PCR.

Commonly misidentified lines
(See ICLAC register)

No commonly misidentified cell lines were used.

Animals and other organisms

Policy information about studies involving animals; ARRIVE guidelines recommended for reporting animal research

Laboratory animals

For in vivo mouse model, 8-12-week-old female NOD.Cg-Prkdc<scid>/Jmice weighing 19-25 g were obtained from the University of Maryland at Baltimore. Animal rooms were maintained as follows: 12/12 light cycles, 30-70% relative humidity and temperature between 20 -25.5°C. Mice were housed in individually ventilated cage/rack systems. Cages were changed at least every 2 weeks. Cages/lids were sanitized and contained corncob bedding and food pellets, which were autoclaved. Fresh Hydro-pac pouches and lickers were placed in cages at time of animal transfer utilizing a Biosafety cabinet. Mice were fed Teklad Global 18% Protein Extruded Rodent Diet.

For Ex Ovo chick embryo cancer xenograft model, fertilized White Leghorn chicken eggs were obtained from University of Alberta Poultry Research Centre.

Wild animals

This study did not involve wild animals.

Field-collected samples

This study did not involve samples collected from the field.

Ethics oversight

All mouse experiments were performed in accordance with the Institutional Animal Care and Use Committee procedures and guidelines of the University of Maryland at Baltimore under approved protocol number 0219006. All procedures involving chick embryos were approved by the University of Alberta Institutional Animal Care and Use Committee (IACUC).

Note that full information on the approval of the study protocol must also be provided in the manuscript.
